# Immunological response and tissue loss in a rodent model of chronic traumatic brain injury treated with resolvin

**DOI:** 10.1186/s12950-025-00471-9

**Published:** 2025-10-02

**Authors:** Olivia Kiwanuka, Julie Cheung, Anders Hånell, Caroline Lindblad

**Affiliations:** 1 Department of Clinical Neuroscience, Karolinska Institutet, Stockholm, SE-171 77 Sweden; 2 Department of Surgery, Södersjukhuset, Stockholm, Sweden; 3 Department of Medical Sciences, Uppsala University, Uppsala, Sweden; 4 Department of Neurosurgery, Karolinska University Hospital, Stockholm, Sweden

**Keywords:** Resolvin, Traumatic brain injury, Preclinical, Neuroinflammation, Rodent

## Abstract

**Background:**

Traumatic brain injury (TBI) triggers neuroinflammation both acutely and chronically, the latter which might be involved in neurodegenerative disorders. Resolvins are neuroinflammatory modulators, hypothesized to improve resolution of inflammation. This study sought to explore sustained immunological responses after delayed treatment with resolvins utilizing novel tools for automated cellular assessments.

**Materials and methods:**

Twenty-five rodents (Sprague-Dawley rats) were exposed to a penetrating TBI, following which delayed treatment with resolvin was initiated. Assessments of tissue loss, and persistent neuroinflammatory activation, was assessed at 6 weeks post-injury utilizing histological and immunohistochemical methods. We also developed a novel computational tool to count and automatically assess cell counts across treatment groups.

**Results:**

The TBI model elicited a substantial brain injury, as expected. The lesion cavity volume was not affected by resolvin or vehicle treatment. Notably, both microglial and macrophage responses were also similar between treatment groups, as deemed by state-of-the-art computational models.

**Conclusion:**

Resolvins administered in a delayed fashion following severe TBI did not affect the extent of chronic microglial or macrophage responses, but warrants future corroboration. Dosing and timing of resolvin treatment warrants further study.

##  Introduction

 Traumatic brain injury (TBI) initiates a complex cascade of cellular and molecular events that extend well beyond the acute phase and may culminate in chronic neuroinflammation and progressive tissue loss [1–3]. Beyond the immediate mechanical damage, TBI initiates a complex secondary injury cascade characterized by neuroinflammation, oxidative stress, and neuronal cell death [3, 4]. Modulating this secondary phase holds promise for improving outcomes, yet effective pharmacological interventions remain limited.

Specialized pro-resolving lipid mediators (SPMs), derived from omega-3 polyunsaturated fatty acids, play an important role in actively resolving inflammation rather than merely suppressing it [5, 6]. Among these, docosahexaenoic acid (DHA)-derived mediators such as resolvins (RvD), protectins, and maresins have gained increasing attention for their neuroprotective and anti-inflammatory properties. SPMs can reduce leukocyte infiltration, limit cytokine production, and promote tissue repair, offering a distinct approach from traditional anti-inflammatory therapies [7]. However, the efficacy of delayed administration of these mediators in mitigating long-term outcomes after TBI remains largely unexplored.

Recent preclinical studies have highlighted the therapeutic potential of resolvins in the acute and subacute phases of TBI. Ren et al. demonstrated that Resolvin D1 (RvD1), administered shortly after injury, ameliorates cognitive impairment via protection of astrocytic mitochondria, a process critical for maintaining energy homeostasis and limiting reactive oxygen species generation [8]. Similarly, Thau-Zuchman and colleagues reported that a single injection of DHA induced a pro‐resolving lipid mediator profile within injured tissue, resulting in a long‐lasting reduction in neurological deficits in mice [9]. Treatment with RvD in murine models led to a dose-dependent reduction in leukocyte infiltration [5], and both pre- and post-injury administration of resolvins increased the presence of ramified microglia in a mouse TBI model [10].

Despite these promising insights, significant gaps remain in our understanding of how delayed therapeutic interventions with resolvins impact long-term histopathological and functional outcomes. Traditional histological assessments are labor-intensive and prone to observer bias, limiting high‐throughput analysis of cellular responses across extended time points. To address this challenge, we have developed a novel automated method for quantitative cellular assessment, enabling reproducible measurement of immune cell activation with minimal user intervention.

This study aimed to explore sustained immunological responses and ongoing tissue loss following experimental TBI. Specifically, we sought to investigate whether delayed treatment with the resolvin RvD1 could affect chronic tissue loss or inflammation, as assessed using a novel automated cellular assessment technique. By integrating advanced image analysis with targeted lipid mediator therapy, we aimed to elucidate the potential of pro-resolving strategies to promote resolution of inflammation and preserve neural tissue long after the initial injury.

##  Materials and methods

### Experimental design

**Fig. 1:**
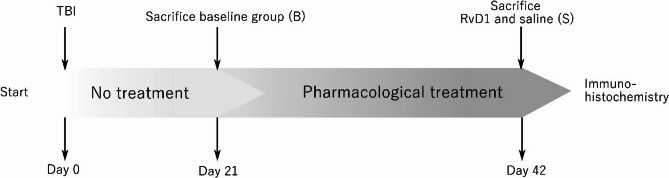
Experimental design. Twenty-five adult male Sprague-Dawley rats underwent a penetrating TBI procedure and were subsequently divided into three groups: resolvin D1- treated (RvD1), saline-treated (S), and a baseline group sacrificed pre-treatment (B). Group B was sacrificed three weeks post-injury while groups RvD1 and S were sacrificed six weeks post-injury. Abbreviations: B, baseline; RvD1, Resolvin D1-treated; S, saline

All experimental procedures adhered to ethical standards as well as Swedish laws and regulations and were approved by the Swedish Board of Agriculture (the local ethics committee) (Approval No. N244/15). Twenty-five adult male Sprague-Dawley rats (10–15 weeks old, 450–650 g) were housed in pairs under a 12-hour light/dark cycle with food and water available *ad libitum*. They underwent a penetrating TBI procedure and were subsequently divided into three groups: resolvin D1- treated (RvD1), saline-treated (S), and a baseline group sacrificed pre-treatment (B). Group B was sacrificed three weeks post-injury while groups RvD1 and S were sacrificed six weeks post-injury. Fig. 1 outlines the experimental design.

### Penetrating traumatic brain injury

We adopted a modified version of the penetrating TBI model from Plantman et al. [11]. This is one of few penetrating TBI models suitable for rodent work [12]. Within the model, a reproducible TBI with high degree of animal survival is accomplished via a probe that penetrates the brain at a standardized depth (typically ~ 5 mm). The probe is accelerated via a lead pellet, in turn propagated via an air pressure from a rifle. The probe depth is limited via a probe holder [11, 12]. Variations and severity alterations to the model can be achieved via probe velocity, probe shape, and probe penetration depth [12, 13]. The major limitation of the model is that injuries can only be achieved in one hemisphere in the coronal plane [12]. This limits translational potential with for example bi-hemispheric injuries.

Here, animals were anesthetized using isoflurane (4% induction, 2% maintenance) and placed in a stereotaxic instrument. Buprenorfin 50 mg/g subcutaneously (sc) was administered prior and after the surgery and Lidocaine 0.1 ml was administered sc in the scalp before incision. During the entire procedure body temperature was controlled with a thermostatically regulated heating pad, while heart rate and arterial oxygen saturation were monitored using puls-oxymetry attached to the hind leg. A 3 cm long midline incision was made through the skin and periosteum to expose the skull and a 2 mm-diameter burr hole was drilled, using a dental drill, 2 mm lateral and 2 mm caudal of bregma. One of twenty-five rats died from the surgery prior to injury. The animals were then positioned with the impactor probe directly above the dura that had been exposed by the burr hole. The penetration injury was acquired through a lead pellet that was accelerated by air pressure from a modified air rifle. None of the animals died from the injury. The incision was thereafter closed with resorbable sutures (size 3.0) and the animals were permitted recovery time in a single cage before reunited with their cage mates.

### Pharmacological treatment

Three weeks post-TBI the 24 animals that survived surgery were randomly divided into 3 groups with *n* = 8 animals in each. The baseline group (B) was immediately sacrificed for baseline data collection. The remaining groups received weekly intraperitoneal injection of either sterile saline (S) or RvD1 (RvD1) (Cayman Chemical, US, Item No. 10012554, 10 µg) for 3 consecutive weeks before sacrifice. RvD1 was handled in order with the manufacturer’s instruction [14]. RvD1 was delivered in ethanol solution, from which aliquots were prepared by further diluting the RvD1 using saline. Upon a planned injection, RvD1 was thawed to room temperature prior to injection. The stability of the substance can be affected by light, repeated freeze/thaw cycles, and specific solvents, according to the manufacturer [14]. In addition, RvD1 is sensitive to enzymatic degradation [5].

The dosing of RvD1 (10 µg per weekly injection) was based on other studies of SPM administration, including previous studies using RvD1. Very few, if any, studies have examined RvD1 in the chronic phase after CNS injury. Notably, the dose administered was in the higher interval, because of the chronic time point, which yields larger experimental animals. RvD1 (or aspirin-triggered RvD1) has been administered in doses ranging from 30 ng to 2 µg at varying intervals [8, 10, 15–20]. Several of these studies involved various dosing regimens and administration routes as well as different injury models, both traumatic and non-traumatic. Other studies investigating other SPMs have used substantially higher doses [21–23], and some lower doses [24]. The timing of dosing was decided to intervene in the chronic phase of traumatic brain injury [25].

A core aspect of RvD1 function is its effect on neutrophils [5]. As we sought to study the chronic phase after TBI this was unfeasible to use for assessment of biological RvD1 activity as we would have expected the post-TBI neutrophil response to occur much earlier than our study end time [25]. In previous work (not exclusively on TBI), some authors have assessed the endogenous and compiled endogenous and exogenous levels of RvD1 using e.g. ELISA [15, 17, 18]. Others have refrained from this and exclusively used indirect measures of RvD1 activity [8, 10]. The latter was similar to our approach, where we did not use any specific quantitative assay to establish the intracerebral RvD1 concentrations.

### Tissue preparation

Rats were trans-cardially perfused with phosphate-buffered saline (PBS) for 5 min followed by 4% paraformaldehyde for 10 min. The whole brain was harvested, placed in 30% sucrose for 24 h and thereafter flash frozen in dry ice and stored in −70 °C until coronal sections were cut using a cryostat (Cryo-Star HM 560 M, MICROM International, Walldorf, Germany). Fourteen micrometer’s thick coronal sections were collected from six equally spaced bregma levels spanning the lesion area. Sectioning started at bregma level + 1 (the coronal plane 1 mm anterior to the bregma) and ended at bregma level − 4 (the coronal plane 4 mm posterior to the bregma), with ten sections collected from each level.

### Hematoxylin and eosin

Hematoxylin and eosin staining (HES) was used for analyzing tissue loss. The sections were rehydrated in PBS for 60 s and then in deionized water for 10 s. They were then stained with hematoxylin (Harris HTX) for 17 min, rinsed with tap water for 5 min and then de-stained in acid ethanol for 30 s. After rinsing with tap water, the sections were immersed in ammonion hydroxide (Merck KGaA) for 45 s and then in warm water for 60 s. After dipping them into ethanol for 10 s they were stained with eosin (HistoLab) for 15 s before dipped in deionized water for 30s. The slides were dehydrated with ethanol and xylene during a total of 50 min. Cover slips were applied with Entellan (Merck KGaA).

### Immunohistochemistry

To assess the immunological response, sections were stained with immunohistochemistry for immune cells. Frozen sections were thawed to room temperature and outlined using a PAP pen. They were then rehydrated in 0.01 M PBS (Sigma-Aldrich) for 30 min. The sections were then incubated over night at + 4 °C with antibodies Anti-Iba1 (1:500, FUJIFILM Wako Pure Chemical Corporation, 019–19741), ED1/CD68 (1:1000, BIO-RAD, MCA341R), MPO (1:50, Jackson ImmunoResearch, GR325073-3), and CY3/CD3 (1:50, Jackson ImmunoResearch, GR293692-3).

The following morning the sections were washed in 0.01 M PBS and thereafter incubated with secondary antibodies: Donkey Anti-Mouse IgG (1:400, Jackson ImmunoResearch, 715-165−151), and Donkey Anti-Rabbit IgG (1:400, Jackson ImmunoResearch, 711-545−152) for 2 hours in an opaque incubation box at room temperature. Cell nuclei were stained with DAPI (4’,6-diamidino-2-phenyl-indole). The sections were then cover-slipped using 70 µl Mowiol (Sigma-Aldrich) as mounting medium.

### Imaging

Images of each section were captured using a confocal microscope with an attached digital camera (Nikon, H550L, Japan, for HES; Nikon, Andor Zyla, for IHC). We took high-resolution (2GB per image) images of the entire section. The excitation fluorescence filters GFP and Texas red were used, with an exposure time of 100 milliseconds. To obtain a large image, five smaller parts of the section were scanned on the width and six lengthwise, for a total of 30 images, at 10X magnification. These were then stitched together using the software NIS-Elements (Japan). The images were acquired in 12-bit grey scale and converted to 8-bit RGB image when saved.

### Image analysis

#### Tissue loss assessment

The brain tissue volumes were calculated with the software SectionToVolume using the Cavalieri principle [26]. This method hinges on the software’s ability to distinguish tissue from the background by analyzing pixel color. Prior to volume estimation, the pixel size is defined, enabling the software to calculate area measurements through pixel counting. The analysis incorporates data from six bregma levels, spaced equally, with the inter-level distance clearly specified. By assessing the areas of HES stained tissue and cavities, and considering the known distance between sections, the software accurately estimates the volumes of interest.

#### Cell calculations

**Fig. 2:**
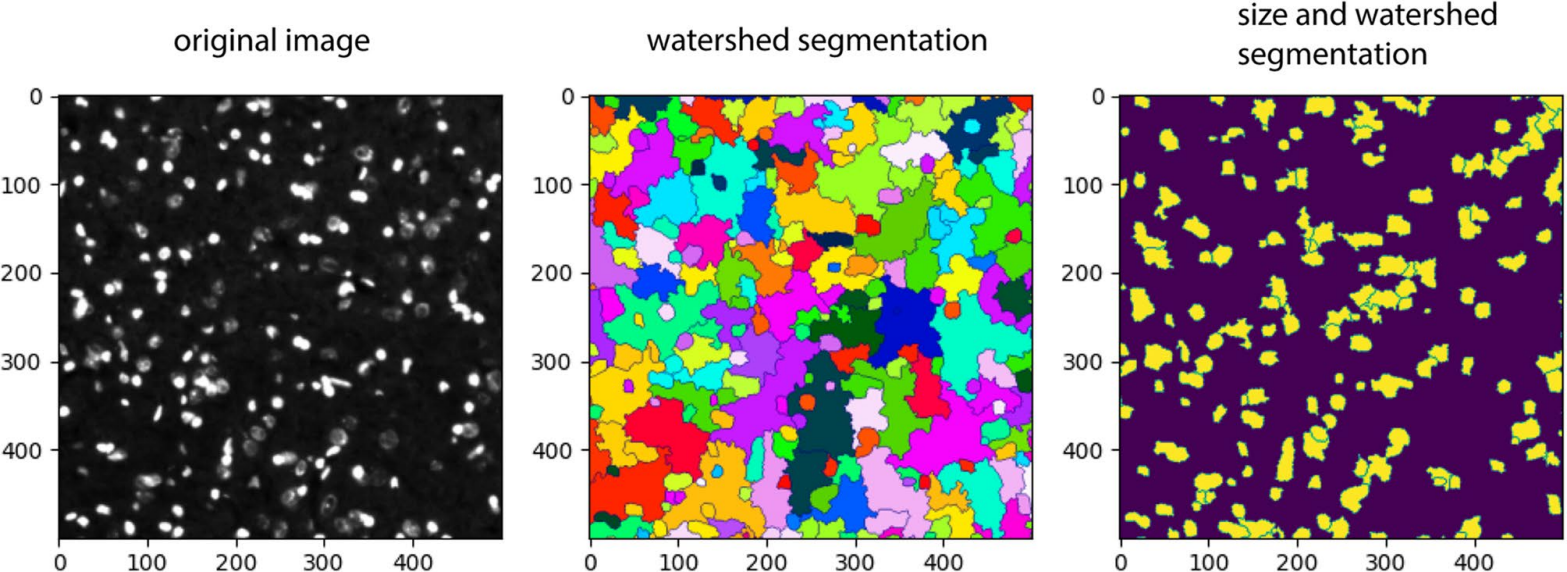
Automated cell count strategy. Representative example of the segmentation algorithm. Here nuclei stained with DAPI is depicted as in the original image (left), following the watershed algorithm (middle), and following application on a size filter on the watershed algorithm (right). Abbreviations: DAPI, 4’,6-diamidino-2-phenyl-indole

Whole-brain section images were converted to TIFF format and thereafter processed in Python using Jupyter Notebooks within Visual Studio Code. The processing involved various Python libraries, including PIL [27], NumPy [28], pandas [29], cv2 [30], matplotlib [31], and scikit-image [32]. Initially, all images were gamma-corrected and converted from 12-bit to 8-bit. A Gaussian blur was applied to each image, followed by watershed segmentation using cv2. Further refinements included size thresholding based on the image data. A representative depiction is showed in Fig. 2.

DAPI, Iba1, and ED1 markers were assessed and found to be of acceptable quality, whereas CY3 and MPO were not, due to staining issues and/or sparsity of stained cells. One coauthor (JC), blinded to the automatic segmentation results, manually counted cells in three cropped images (utilized for cell segmentation development) per staining. These manual counts were then compared to the automatic segmentation results. For DAPI, the absolute mean error was 12.5% (± 7.1%), for Iba1 it was 65.8% (± 5.7%), and for ED1 it was 44% (± 25.6%), based on three images each. These error rates were considered acceptable, but caution is warranted as this was derived from images used for the segmentation model development.

###  Statistical analysis

All data was analyzed using the statistical software package R [33] via RStudio [34]. Normality was assessed using the Shapiro-Wilk test. Technical replicates were summarized per biological replicate by calculating the mean. Depending on the normality and equal variance assumptions, treatment groups were compared using either ANOVA with Tukey’s post hoc test or the Kruskal-Wallis test with Dunn’s post hoc test. A p value ≤ 0.05 was considered significant.

##  Results

### Tissue and cavity volume

**Fig. 3:**
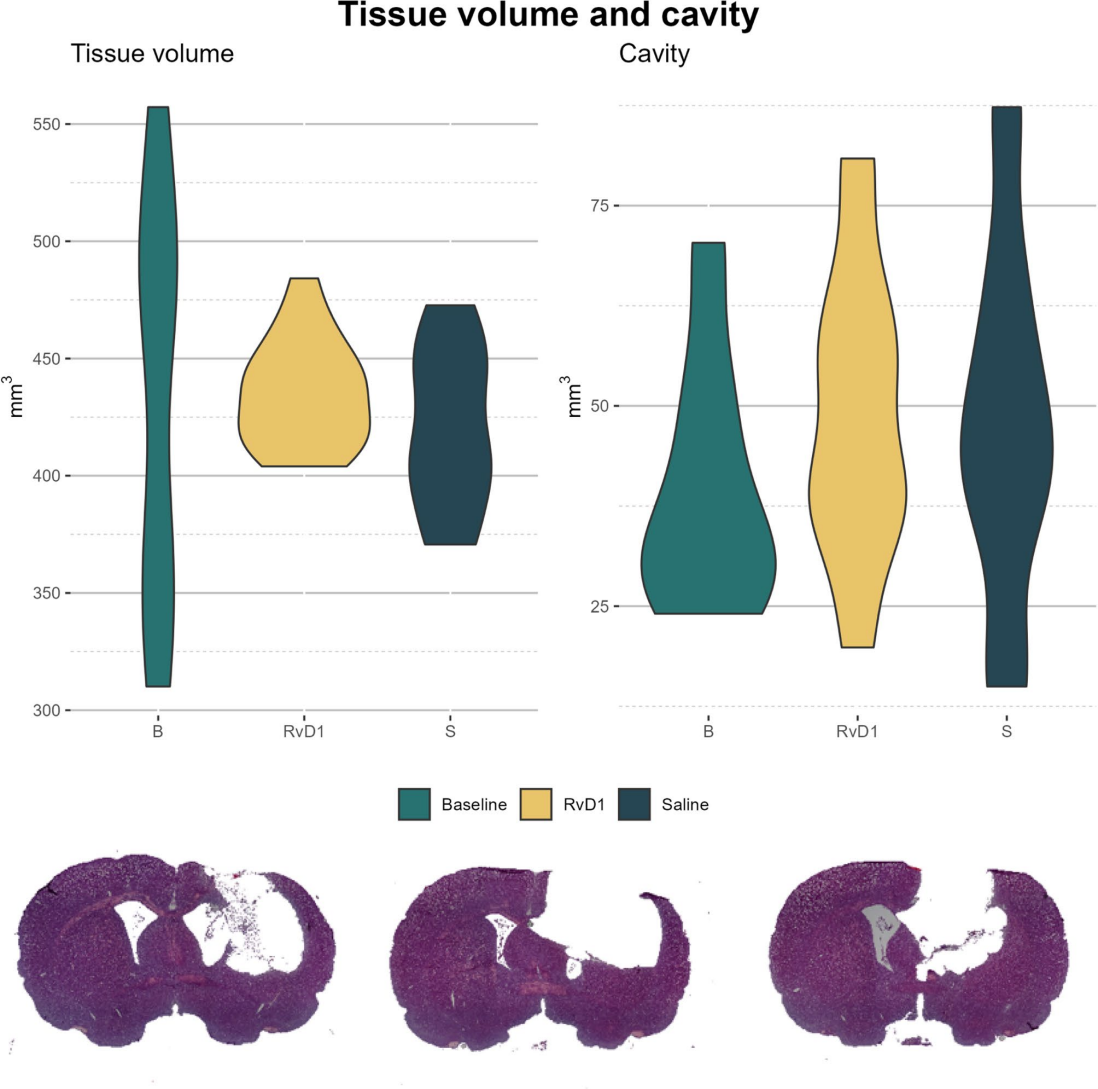
Tissue and cavity volume. Following a penetrating TBI, tissue loss and the consequent lesion cavity were quantified utilizing the software SectionToVolume using the Cavalieri principle [26] (upper panel). In the lower panel, representative HES stainings on which the calculations were based, are demonstrated. Abbreviations: B, baseline; HES, hematoxylin and eosin; R, Resolvin D1-treated; S, saline.

The tissue volume measurements across Baseline, RvD1, and Saline groups showed a median of approximately 500 mm³, 450 mm³, and 450 mm³, respectively, with a wide interquartile range observed within group B (Table 1; Fig. 3). As for the cavity volume resulting from the TBI, medians were approximately 50 mm³ for all groups, with the RvD1 group demonstrating slightly lower variability (Fig. 3). The quantitative analysis of brain tissue and cavity volumes revealed no statistically significant differences between the chronic groups (RvD1/S), nor compared to B (Fig. 3).

**Table 1:** Quantifications of histological tissue loss following TBI

	B	RvD1	S	*p*-value
*n*	8	8	8	
Tissue volume	474 [357, 492]	434 [417, 448]	411 [397, 447]	0.55
Cavity	33 [28, 50]	47 [38, 58]	45 [37, 59]	0.29

Following a penetrating TBI, tissue loss and the consequent lesion cavity were quantified utilizing the software SectionToVolume using the Cavalieri principle [26]. *Abbreviations*: *B* Baseline, *RvD1* Resolvin D1-treated, *S* Saline

### Immunological responses

**Fig. 4:**
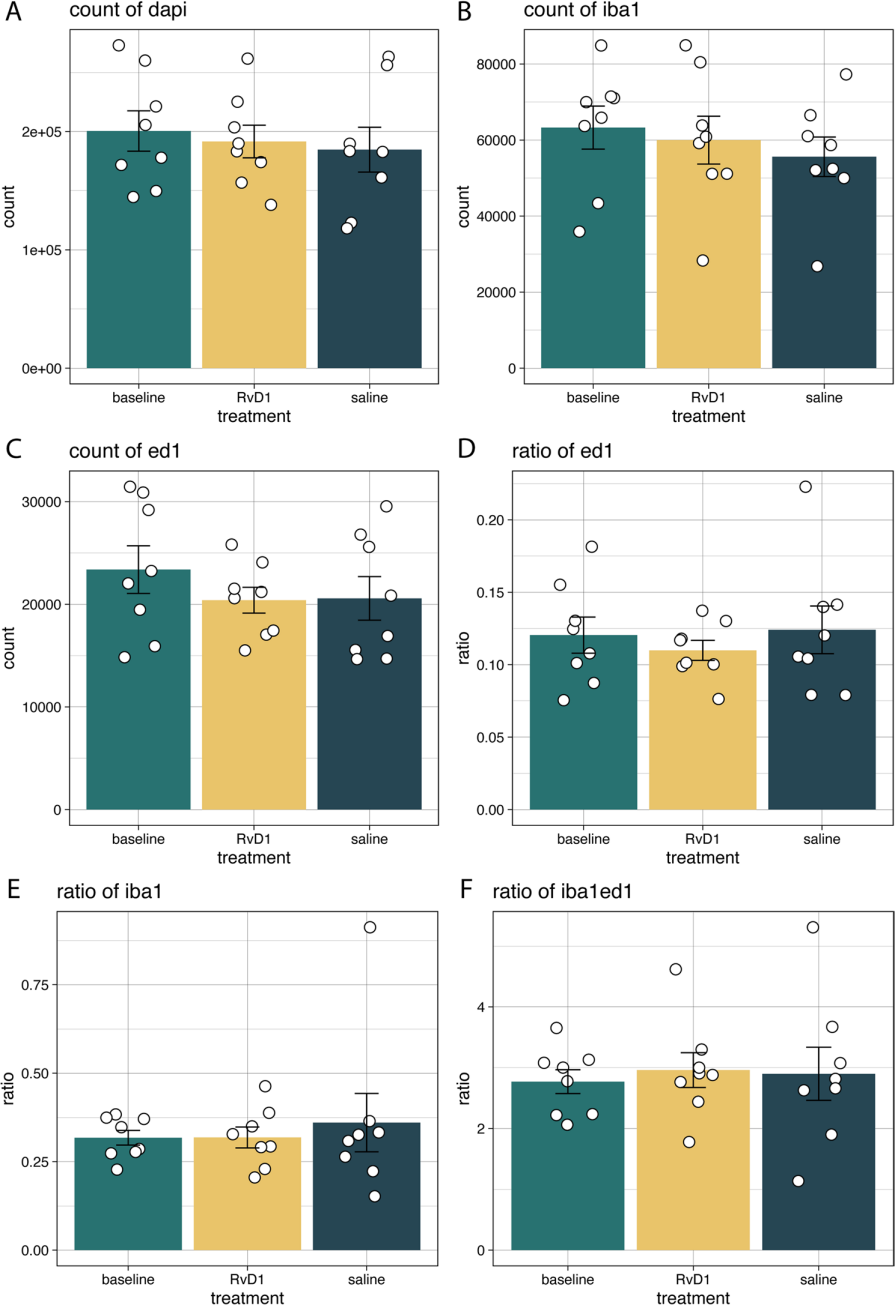
Graphical depictions of whole-brain section quantifications. Cell counts were roughly the same across all treatment groups (**A**, count of DAPI). Neither count nor ratio of Iba1 or ED1 were different across the groups (**B**-**E**). We also assessed the relative relationship between Iba1 and ED1, without any graphical trends or numerically significant findings (**F**). The bar graph denotes treatment group means, whereas each dot represents n = 1 animal. The error bar denotes upper and lower limit of the standard error of the mean. Abbreviations: DAPI, 4’,6-diamidino-2-phenyl-indole; ED1, macrophage marker; RvD1, resolvin D1-treated

In total, *n* = 24 animals had one or two technical replicates subjected to whole-brain section immunohistochemistry assessments (Fig. 4). The total number of cells were roughly the same across all treatment groups (Fig. 4 A). Notably, there was no difference in the number or relative ratios of microglia versus macrophages at 6 weeks following injury (Fig. 4B-E). Accordingly, the relative ratio of microglia to macrophages were also similar (Fig. 4 F). No statistical differences were discerned between the groups with regard to counts and/or ratios of neuroinflammatory cells (Fig. 4).

##  Discussion

In this study we investigated the impact of delayed administration of RvD1 on chronic tissue loss and sustained neuroinflammation in a rodent penetrating TBI model. Our findings demonstrate that initiating weekly intraperitoneal RvD1 injections three weeks post-injury failed to significantly alter tissue or cavity volumes at six weeks compared to saline controls. Immunological markers of ongoing neuroinflammation with microglia activation remained unchanged between groups, indicating limited efficacy of delayed RvD1 treatment on established pathological sequelae. These findings suggest that once chronic neuroinflammation is established, therapeutic modulation may be more challenging than in the acute or subacute phases.

Persistent neuroinflammation is now recognized as a central feature of TBI pathophysiology, extending months to years after the initial insult [4, 35, 36]. Neuroinflammation has a dual role, where early inflammation can promote repair and regeneration, but prolonged activation of microglia and astrocytes contributes to ongoing neurodegeneration, impaired synaptic plasticity, and cognitive decline [2, 3, 37, 38].

SPMs have shown promise in promoting functional recovery, tissue loss, and even as biomarkers for injury severity [9, 15, 38–40]. Our findings contrast with previous research and there are several possibilities for this discrepancy. First, we had a delay of 21 days post trauma before initiating treatment, compared to the acute treatment [8–10, 15]. Our time point was chosen to target the chronic phase of the neuroinflammation, which has not been done previously and has been suggested as a future target of experimental TBI research [41, 42]. The lack of improvement may be because resolvin affects pathways which form part of the acute phase, and has limited effect on the chronic phase. Another consideration is the dosage and administration method, which varies widely. Many studies use intraperitoneal injections, like we did [8, 10, 15], others intravenous [9], but some gave daily treatments [8, 10], every other day [15] or a single injection [9]. It is also possible that the total dosage of RvD1 did not suffice for measurable effect. A third possibility is that our histological endpoints, such as lesion volume and immune cell density, while robust, may be too coarse to capture subtle neuroprotective or neurorestorative changes induced by the treatment. Future studies incorporating molecular markers, electrophysiological measures, or functional behavioral assessments may be better to detect treatment effects.

A key strength of this study is the application of a novel automated image analysis pipeline for quantifying histological markers of tissue loss and neuroinflammation. Traditional manual counting methods are inherently limited by observer bias, inter-rater variability, and time-intensive processing, which constrain throughput and reproducibility [43]. By leveraging computational segmentation algorithms tailored to fluorescent cell nuclei labelling and immunohistochemical markers, our automated approach provides objective, reproducible, and high-resolution quantification at scale. This methodology is particularly relevant for chronic TBI studies, where subtle, spatially heterogeneous changes occur over extended periods. Automated quantification facilitates unbiased comparisons across treatment groups and time points, enabling more nuanced evaluation of cellular responses. Future refinements may incorporate machine learning classifiers to distinguish microglial morphological phenotypes and astrocytic reactive states, improving resolution of functional heterogeneity within CNS immune responses.

### Strengths and limitations

The main strength of this study lies in its novelty, as it is the first to investigate delayed administration of RvD1 in the chronic phase of TBI using an automated, high-throughput histological analysis method. This approach minimized observer bias and allowed for objective quantification of immune cell activation, strengthening the reliability of the findings. The therapeutic concept was grounded in the biology of endogenous inflammation resolution, adding a strong mechanistic rationale.

However, several limitations must be acknowledged. The bioactivity of RvD1 was not confirmed prior to administration. While some studies have assessed RvD1 concentration using e.g. ELISA [15, 17, 18], others have limited their work to merely include indirect measures of RvD1 activity [8, 10]. Since our work focused on the chronic phase after TBI, which has been but sparsely studied, intracerebral concentration measurements of RvD1 would have strengthened the negative results. The study employed a single-dose, single-time-point design, which limits conclusions about the optimal timing and dosing regimen for intervention. Since the chronic neuroinflammatory phase of TBI has not been investigated before, both dose-response assessments of RvD1 as well as investigations of optimal treatment timing would have increased the robustness of our results. In fact, earlier intervention might be necessary to effectively modulate the chronic inflammatory processes observed in TBI, as the delayed treatment could have missed the most crucial therapeutic window. Yet, our data provides a first step for chronic TBI studies investigating resolution of inflammation, and might thus be an important addition to the sparse literature on the topic despite its negative results.

The observed variability in tissue and cavity volumes, indicated by wide interquartile ranges, exemplifies the diversity of the injury model. Together with the limited sample size, this might have caused a type II error, which could have contributed to the absence of statistically significant findings. Lastly, the study’s reliance on tissue volume and cavity size as primary metrics for assessing chronic TBI may not fully capture the complexities of chronic neuroinflammation and tissue damage. Incorporating additional markers and methods, such as biochemical assays for inflammatory mediators in tissues and biofluids, single cell RNA sequencing, or advanced imaging techniques, could provide a more comprehensive understanding of chronic TBI pathology and the efficacy of treatments like RvD1.

##  Conclusion

In this rodent study, delayed treatment with RvD1, initiated 21 days post TBI, did not yield significant effects on tissue volume or inflammatory cell counts compared to saline-treated controls at 6 weeks post-injury. The current study is small, and investigates limited aspects of resolution of inflammation in the chronic phase after TBI, why results should be interpreted cautiously. Yet, this scalable method offers a promising tool for future research, enabling a more comprehensive assessment of chronic inflammatory responses following experimental TBI.

## Data Availability

The datasets and/or code used are available upon request from the corresponding author.

## Abbreviations

B: Baseline-treated (control) group
BBB: Blood-brain barrier
CNS: Central nervous system
DAMP: Damage-associated molecular pattern
DAPI: 4’,6-diamidino-2-phenyl-indole
DHA: Docosahexaenoic acid
EPA: Eicosapentaenoic acid
HES: Hematoxylin and eosin staining
PBS: Phosphate-buffered saline
RvD: D-type resolvins
RvD1: Resolvin D1-treated
RvE: E-type resolvins, RvE
ROS: Reactive oxygen species
S: Saline-treated (vehicle-treated) groups
SPM: Specialized pro-resolving lipid mediators
Sc: Subcutaneously
TBI: Traumatic brain injury
